# Antigen Production in Plant to Tackle Infectious Diseases Flare Up: The Case of SARS

**DOI:** 10.3389/fpls.2016.00054

**Published:** 2016-02-05

**Authors:** Olivia C. Demurtas, Silvia Massa, Elena Illiano, Domenico De Martinis, Paul K. S. Chan, Paola Di Bonito, Rosella Franconi

**Affiliations:** ^1^Department of Sustainability, Biotechnology Laboratory, Italian National Agency for New Technologies, Energy and Sustainable Economic DevelopmentRome, Italy; ^2^Department of Sustainability, Biomedical Technology Laboratory, Italian National Agency for New Technologies, Energy and Sustainable Economic DevelopmentRome, Italy; ^3^Department of Pharmacological and Biomolecular Sciences, Università degli Studi di MilanoMilan, Italy; ^4^International Relations Office, Italian National Agency for New Technologies, Energy and Sustainable Economic DevelopmentRome, Italy; ^5^Department of Microbiology, Faculty of Medicine, The Chinese University of Hong Kong, Prince of Wales HospitalHong Kong, China; ^6^Centre for Emerging Infectious Diseases, Faculty of Medicine, The Chinese University of Hong Kong, Prince of Wales HospitalHong Kong, China; ^7^Istituto Superiore di Sanità, Department of Infectious, Parasitic and Immune-Mediated DiseasesRome, Italy

**Keywords:** SARS-CoV, severe acute respiratory syndrome (SARS), N protein, M protein, plant expression, disease outbreaks, emerging infectious disease

## Abstract

Severe acute respiratory syndrome (SARS) is a dangerous infection with pandemic potential. It emerged in 2002 and its aetiological agent, the SARS *Coronavirus* (SARS-CoV), crossed the species barrier to infect humans, showing high morbidity and mortality rates. No vaccines are currently licensed for SARS-CoV and important efforts have been performed during the first outbreak to develop diagnostic tools. Here we demonstrate the transient expression in *Nicotiana benthamiana* of two important antigenic determinants of the SARS-CoV, the nucleocapsid protein (N) and the membrane protein (M) using a virus-derived vector or agro-infiltration, respectively. For the M protein, this is the first description of production in plants, while for plant-derived N protein we demonstrate that it is recognized by sera of patients from the SARS outbreak in Hong Kong in 2003. The availability of recombinant N and M proteins from plants opens the way to further evaluation of their potential utility for the development of diagnostic and protection/therapy tools to be quickly manufactured, at low cost and with minimal risk, to face potential new highly infectious SARS-CoV outbreaks.

## Introduction

Recombinant proteins expressed in plants have emerged as a novel branch of the biopharmaceutical industry and hold great potential to produce different types of therapeutic proteins at low cost and with reduced risks of contamination with human and animal pathogens (Moustafa et al., 2015; Paul et al., 2015; Sack et al., 2015). Transient expression of target proteins can be easily achieved by plant viruses or by agroinfiltration (Gleba et al., 2007), saving the time spent in the generation of transgenic plants, often allowing higher protein yield due to the absence of chromosomal integration and consequently of position effects (Komarova et al., 2010). Transient expression can also be used as a means for preliminary evaluation of correct expression before starting the generation of transgenic plants, or related platforms, such as plant cell cultures or microalgae (Franconi et al., 2010).

Antigen preparation plays a crucial role in the development of a diagnostic test, and plants represent an ideal biofactory system. The approach could be extended to other cases when a pathogen cannot be grown in the lab or is highly virulent and needs a methodology for fast and affordable production. Indeed, virus-specific and ‘orphan’ vaccine candidates and therapeutics represent one of the most interesting applications of the plant-based technology, especially when it is necessary to produce ‘rapid response’ vaccines such as those directed against bioterrorism agents and diseases with pandemic potential, like influenza. The capacity for such a response has already been demonstrated by the (four) companies involved in the US in the production of 100 million doses of influenza vaccine a month (Rybicki, 2014). This opens the way for the use of this technology for other diseases such as, to name a few, Ebola (Henao-Restrepo et al., 2015; Heymann et al., 2015), avian flu (Su et al., 2015), MERS (Al-Tawfiq and Memish, 2015; Keener, 2015) and other viruses where the principles of the so-called “One Health Initiative” (strategies to control diseases across species) are important (http://www.onehealthinitiative.com/). The fact that the epidemiology of these diseases is associated to sudden and sometimes unforeseen contagious burst, results in an on/off attention about, in terms of research, prevention and pharma industry efforts (Barber, 2014; LaMattina, 2014). For this reason, tools for a quick and relatively easy scale-up may provide a solution to such emergencies (Streatfield et al., 2015).

Among emerging and re-emerging diseases, severe acute respiratory syndrome (SARS) appeared in China in November 2002. The epidemic spread to 29 countries over 5 continents, leading to more than 8000 infected patients globally (World Health Organization [WHO], 2006) with a fatality rate of 9.6% (Suresh et al., 2008). The aetiological agent of the syndrome, the coronavirus SARS-CoV, was rapidly identified (Drosten et al., 2003; Marra et al., 2003; Rota et al., 2003). The end of the SARS outbreak was declared by World Health Organization [WHO] (2003) in July, thanks to strong containment measures. However, several local outbreaks were subsequently reported in China, as a consequence of accidental laboratory contaminations or infections after contact with animals infected with SARS-CoV strains significantly different from those predominating in the 2002–2003 outbreak (Peiris et al., 2004). While no effective therapy is currently available, considerable efforts have been made to develop vaccines and drugs to prevent SARS-CoV infection, since a SARS epidemic may recur at any time in the future (Gimenez et al., 2009; Chan and Chan, 2013). Moreover, because of the highly contagious nature of the disease, and since SARS-CoV has been defined a potential biological weapon (Centers for Disease Control and Prevention [CDC], 2012), it is still important to develop effective SARS-CoV sensitive diagnostic tools (Suresh et al., 2008).

The large SARS-CoV genome, a polyadenylated RNA of 29,727 nucleotides, encodes four major viral structural components, the spike (S), envelope (E), membrane (M), and nucleocapsid (N) proteins, and 16 non-structural proteins (Bartlam et al., 2007).

The structural N protein is the most abundant protein in the SARS-CoV virion. It is a highly basic protein of 422 amino acids (46 kDa) of the helical nucleocapsid, playing an important role in viral pathogenesis, replication, RNA binding, cell cytokinesis and proliferation (Surjit and Lal, 2008). N protein has been recognized as the preferred target for detection of SARS-CoV infection by reverse transcription-polymerase chain reaction (RT-PCR; Suresh et al., 2008). In addition, the WHO guidelines for SARS diagnosis, developed during the outbreak in 2003, suggested the use of N-based ELISA for specific IgG detection as confirmatory test of SARS-CoV infection (World Health Organization [WHO], 2003 SARS: Laboratory diagnostic tests) due to the ability of the host to mount an early antibody response against the N protein (Che et al., 2004).

Furthermore, since the N protein is able to induce a long-term cell-mediated immune response in animal models, it represents a potential vaccine candidate as well (Roper and Rehm, 2009). To date, the production of recombinant N protein has been achieved in a variety of heterologous expression systems, including plants, (Zheng et al., 2009), providing proofs of concept for its use in vaccine formulations (Roper and Rehm, 2009). However, the immune response in animal models (both natural and non-natural SARS-CoV hosts) might be not useful to predict the human immune response.

The M protein is the most abundant protein in the SARS-CoV viral envelope. It is functionally involved in the assembly and budding of virions from the cell. M protein forms homo-oligomers and interacts with S, E, and N proteins (Hogue and Machamer, 2008). It consists of 221 amino acids (25 kDa), with a short glycosylated N-terminal domain, three membrane-spanning domains and a long immunogenic C-terminal cytoplasmic domain. It has been reported that rabbit antiserum raised against recombinant M protein produced in yeast has a potent neutralizing activity *in vitro* (Pang et al., 2004). Antibodies to the M protein were detectable in convalescent SARS patients and B-cell epitopes of the M protein have been identified (He et al., 2005). It has also been shown that M acts as a dominant immunogen for CTL response in humans (Liu et al., 2010). Moreover, it has been demonstrated that SARS-CoV M-specific memory CD4+ and CD8+ T cells were persistent in the peripheral blood of recovered SARS patients more than 1 year after infection (Yang et al., 2007). In a study, where different DNA vaccines were used, M generated the strongest T- cell response in an animal model, and recovered SARS patients had a long-lasting CD4+ and CD8+ memory for the M antigen (Roper and Rehm, 2009). These data suggest that further research should be directed toward evaluating the potential efficacy of the M antigen for vaccine and diagnostic tools development.

In this study, we demonstrate the feasibility of using plant transient expression systems (Potato Virus X [PVX]-mediated infection and agroinfiltration) to produce two SARS-CoV antigens, the N and M proteins, as useful tools to face SARS-CoV infection.

In particular, we demonstrated that the SARS-CoV N protein produced in *Nicotiana benthamiana* is recognized by the specific antibodies of convalescent SARS patients. Moreover, the expression of the SARS-CoV M protein was achieved for the first time in plant.

The approach to rapidly get crucial antigens by transient expression in plants, potentially attains to other infectious, either emerging or re-emerging, diseases (e.g., MERS, Avian flu, Ebola), that share with SARS the features of rapid outbreak burst and need to rapidly produce diagnostic or therapeutic tools.

## Materials and Methods

### Cells

*Escherichia coli* XL1 blue strain was used as a host for cloning and protein expression. Cells were grown in Luria-Bertani medium at 37°C with shaking at 250 rpm. *Agrobacterium tumefaciens* GV3101 and C58C1 strains were used to transiently express the M protein in *N. benthamiana* and were grown in YEB medium (5 g/l beef extract, 1 g/l yeast extract, 5 g/l peptone, 5 g/l sucrose, 2 mM MgSO_4_) at 28°C with shaking at 250 rpm. When necessary, ampicillin (100 μg/ml) or kanamycin (25 μg/ml) was added to the culture medium. HEK-293 cells were cultivated as a monolayer in DMEM medium with 10% fetal bovin serum (FBS) and 50 μg/ml gentamicin at 37°C with 5% of CO_2_ and relative humidity of 94%.

### DNA Manipulation for Bacterial and Plant Expression of the SARS-CoV N and M Proteins

The nucleocapsid (N, GenBank protein id. AAP33707.1) and membrane (M, GenBank protein id. AAP33701.1) full-length genes of the human SARS-CoV Frankfurt I isolate, Acc. No. AY291315 were cloned into pCR2.1-TOPO TA Cloning vector (Invitrogen; Carattoli et al., 2005). The inserted fragments were cut out by digestion with BamHI-NotI and sub-cloned into the pQE-30 (Qiagen) prokaryotic expression vector (pQE-30-N and pQE-30-M).

**FIGURE 1:**
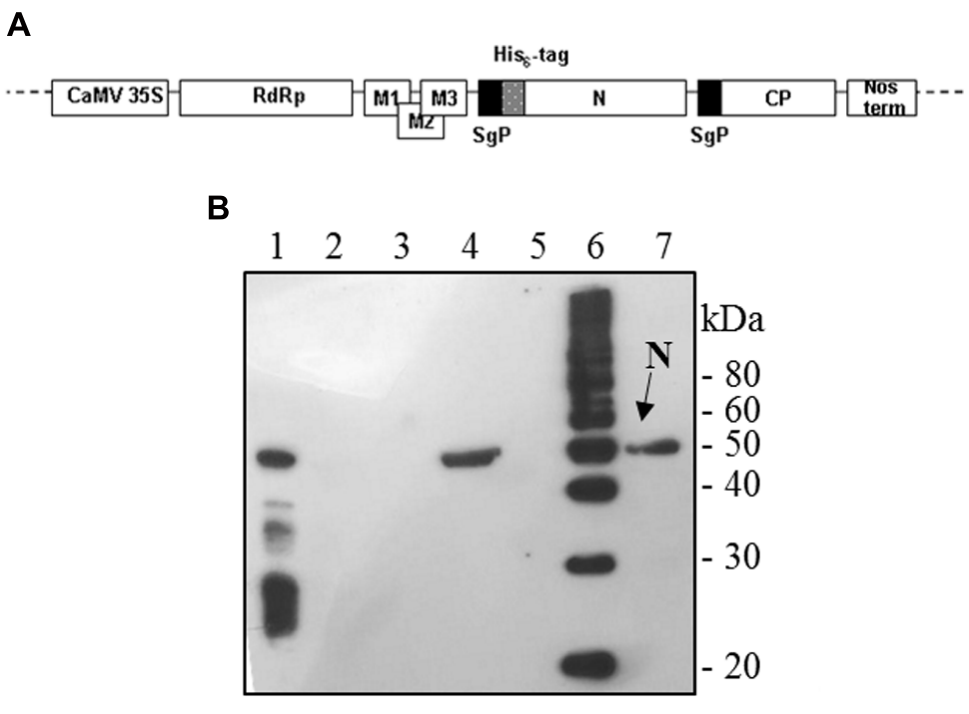
**Potato Virus X-mediated expression of SARS-CoV N protein in *Nicotiana benthamiana* plants. (A)** Schematic representation of the pPVX-N construct used for the expression of SARS-CoV N protein in *N. benthamiana* leaves. CaMV 35S: constitutive 35S promoter from the cauliflower mosaic virus; Nos-ter: transcription terminator of the nopaline synthase gene of *Agrobacterium tumefaciens*; RdRp: PVX replicase gene; M1-3: PVX triple gene block for cell-to-cell movement; CP: PVX coat protein gene; SgP: coat protein sub-genomic promoter; N: SARS-CoV N gene; His_6_-tag: histidine tag. **(B)** Immunoblotting of TSP extracted from *N. benthamiana* plants infected with pPVX-N. For each sample, 20 μg TSP were loaded on gel. Lane 1: N protein purified from *Escherichia coli* under native conditions (40 ng); lane 2: pPVX-N inoculated leaves; lane 3: pPVX201 inoculated leaves (negative control); lane 4: pPVX-N symptomatic systemic leaves; lane 5: pPVX201 symptomatic systemic leaves (negative control); lane 6: molecular weight marker (Magic Mark, Invitrogen); lane 7: pPVX-N symptomatic systemic leaves extract stored at -20° C for 2 months. Immunoblotting was performed with the rabbit anti-N pAb.

The full-length *N* gene (1269-bp long) was amplified by PCR on the template plasmid pQE30-N with *Pfu* polymerase with the forward primer 5′-GGCCATCGAT*GAATTC*GGATCCATC**ATG**AGAGGATCGC ATCACATCC-3′ (*ClaI* restriction site: underlined, *EcoRI* site: italic, initiation translation codon: bold) and the reverse primer 5′-GACTTGTCGAC*GCGGCCGC***TTA** TGCCTGAGTTGAATCAGCAG-3′ (*SalI* site: underlined, *NotI* site: italic, stop codon: bold). For mammalian cells expression, the PCR product was cut out by digestion with *EcoRI/NotI* and inserted into the pVAX1 vector (Invitrogen). For plant expression, the PCR product was cut out with *ClaI/SalI* and cloned into the pPVX201 plant vector (Baulcombe et al., 1995). In this vector, the full-length viral cDNA of the Potato virus X (PVX) is inserted between the constitutive 35S promoter of the cauliflower mosaic virus (CaMV 35S) and the transcription terminator (Nos-term) of the nopaline synthase gene of *A. tumefaciens*, necessary for the regulation of the viral genome expression upon infection with plasmid DNA. Characteristic components of the viral expression vector are the following: viral replicase gene (RdRp); triple gene block encoding protein for cell-to-cell movement (M1-3); viral coat protein gene necessary for encapsidation of viral RNA (CP); coat protein sub-genomic promoter (SgP; **Figure 1A**).

The full-length *M* gene (666-bp long) from the pCR2.1-TOPO TA vector was amplified in two subsequent steps. First, the forward primer 5′-GGCCATCGAT*GAATTC*GGATCCATC**ATG**GCAGACAACGG TACTATTAC-3′ (*ClaI* restriction site: underlined, *EcoRI* site: italic, initiation translation codon: bold) and the reverse primer 5′-*GTGATGGTGATGA TG*CTCGAGTGCCTG**TACTAGCAAAGCAATATT**-3′ (end of the His_6_ tag: italic, end of the *M* gene: bold) were used to add the His_6_ tag at the C-terminus. Subsequently, the same forward primer and the reverse primer 5′-GACTTGTCGAC*GCGGCCGC***TCA**ATG*G*TGATGGTGATGATGCTCG-3′ (*SalI* site*:* underlined, *NotI* site: italic, stop translation codon: bold) were used in order to add restriction sites useful for cloning.

For simplicity, we report the name of the recombinant genes *N-His_6_* and *M-His_6_* as *N* and *M* genes. The purified PCR products were cloned into the *Eco RV*-linearized pBlueScript SK(+; pBS) cloning vector (Stratagene; pBS-N and pBS-M), sequenced for authenticity and sub-cloned by *ClaI-SalI* in the pPVX201 plant vector (pPVX-N and pPVX-M) or by *EcoRI/NotI* in the pVAX1 vector (pVAX-N and pVAX-M). After XL1 blue cells transformation, pPVX-N and pPVX-M plasmid DNAs were purified (maxiprep kit, Qiagen) for plant infection, while pVAX-N and pVAX-M plasmids were purified with endotoxin-free purification kits (Plasmid Maxi kit LPS-free, Qiagen) for mammalian HEK-293 cells transfection.

The *M* gene was also sub-cloned by *XbaI/SalI* from the pBS-M construct into the pBI-121 plant binary vector (Clontech Laboratories, Acc. No. AF485783), obtaining the pBI-M construct. In this construct, the *M* gene is inserted between the CaMV 35S promoter and the NOS terminator. Characteristic components of the vector are: right and left borders (RB, LB), gene for kanamycin resistance (NPT II) under the transcription promoter and terminator of the nopaline synthase gene of *A. tumefaciens* (**Figure 6A**).

The pBI-M construct, obtained by substitution of the *GUS* gene with the *M* gene, was used to transform the GV3101 and C58C1 strains of *A. tumefaciens.*

### Expression and Purification of N and M Proteins in Bacteria

The expression and purification of the N and M proteins were done according to standard protocols (The QIAexpressionist, Qiagen). Since it was previously described that the M protein is toxic in bacteria (Carattoli et al., 2005), culture growth was performed at suboptimal temperature of 30°C. In these conditions, we isolated a clone able to express the M protein in *E. coli*. Characterization revealed that it corresponded to a spontaneously mutated *M* gene, coding for a protein carrying the three substitutions K_13_ > R, F_36_ > L, and I_160_ > V (M_RLV_).

The M_RLV_ and the N proteins were purified by Ni-NTA affinity chromatography in denaturing conditions. For the N protein, when purified in native conditions, imidazole concentration in the wash buffer was 30 mM.

Quantification of the N protein purified in denaturing and native condition was performed by Coomassie stained SDS-PAGE comparing the intensity of the band at 50 kDa to known amount of BSA using a Chemidoc ImageLab system with ImageLab 4.0 software (Bio-Rad).

### Expression of N and M Proteins in Mammalian Cells

HEK-293 cell line was transfected in six wells plates with the pVAX-N or pVAX-M constructs according to standard protocols (Effectene Transfection Reagent, Qiagen). 24 h post-transfection (pt) some wells were added with 12.5 μM of the proteasome inhibitor MG-132. 48 h pt both samples, treated or not with MG-132, were harvested and prepared for protein expression analysis (immunoblotting and immunofluorescence). After three washes with cold phosphate-buffered saline (PBS: 21 mM Na_2_HPO_4_, 2.1 mM NaH_2_PO_4_, 150 mM NaCl, pH 7.2) cells were centrifuged at 800 rpm for 5′, recovered and re-suspended in SDS-loading buffer (10% glycerol, 60 mM Tris-HCl pH 6.8, 0.025% bromophenol blue, 2% SDS, 3% 2-mercaptoethanol) and boiled for 10 min.

For immunofluorescence analysis, HEK 293 cells were grown on multi-chamber glass slides and transfected at 40% of confluency. 24 h post transfection cells were washed three times with PBS, fixed with 4% paraformaldehyde for 10 min and permeabilized with 0.1% Triton X-100 in PBS. Samples were blocked with 5% no-fat dry milk in PBS. For detection of the N and M proteins, cells were incubated with specific polyclonal antibodies (pAb) validated in immuno-cytochemical assay on SARS-CoV infected and previously described (Carattoli et al., 2005). For N protein we used a mouse anti-N pAb, (obtained after immunization of animals with the purified His_6_-N protein produced in *E. coli*) at 1:800 dilution. For the M protein we used a mouse anti-M pAb (obtained by immunizing mice with the recombinant cytoplasmic domain, amino acids 138–222, of the M protein produced in *E. coli*) at 1:400 dilution. Cells were then incubated with a 1:300 dilution of an anti-mouse biotinylated secondary antibody (GE Healthcare) and a 1:50 dilution of Streptavidin-FITC conjugated (Sigma–Aldrich, GmbH, Steinheim, Germany). Nuclei were counter-stained with DAPI (2 μg/ml) in PBS. Slides were examined using a fluorescence “Axiolab Zeiss” microscope (Oberkochen, Germany) interfaced with a Coolsnap CCD camera (Roper Scient., Princeton, NJ, USA).

### Expression of N and M Proteins in *Nicotiana benthamiana*

Two leaves of *N. benthamiana* plants (4 week-old) were dusted with carborundum powder and inoculated with 10 μg of the plasmids pPVX-N, pPVX-M, or pPVX201 (empty vector, negative control) diluted in 100 μl bi-distilled water. Plants were also treated with 100 μl bi-distilled water (mock-infected) for monitoring viral infection symptoms. Plants were grown under 16 h daylight at 22°C into a containment greenhouse (bio-safety level 2) and observed daily for PVX infection signs. Inoculated and symptomatic leaves were harvested and stored at – 80°C until use.

Four week-old *N. benthamiana* plants were infiltrated with *A. tumefaciens* C58C1 and GV3101 cultures harboring the pBI-M construct that had been grown and induced as described (Kapila et al., 1997). Plants were subsequently grown as described and the leaf disks collected 3, 4, 5, 6, 7, and 10 days post-infiltration (dpi) were homogenized with an ultraturrax in three volumes (w/v) of SDS-loading buffer and boiled for 3 min.

To analyze N and M protein accumulation in plant tissues, soluble proteins were extracted from plant material using different buffers, depending on their hydrophobic properties. Crude plant extracts were prepared by grinding the tissue to a fine powder in liquid nitrogen. The powder was re-suspended and homogenized with an ultraturrax in three volumes (w/v) of PBS or, alternatively for the M protein, in one volume (w/v) of GB buffer (100 mM Tris-HCl pH 8.1, 10% glycerol, 400 mM sucrose, 5 mM MgCl_2_, 10 mM KCl, 10 mM 2-mercaptoethanol) containing protease inhibitors (“complete, EDTA-free,” Roche Diagnostics, GmbH, Mannheim, Germany). Tissue homogenates were centrifuged at 4°C, 12,000 × *g*, for 10 min. The supernatant was transferred to a fresh tube and kept on ice (or at 4°C) until use and total soluble protein (TSP) content was estimated by the Bradford assay (Bio-Rad Inc., Segrate, Italy). Pellets were re-suspended in appropriates volumes of SDS loading buffer, and prepared as described in the paragraph below, constituting the insoluble fraction of leaf extracts.

Homogenized tissues of pPVX-N infected plants were also used to inoculate *N. benthamiana* plants to propagate the infectious recombinant PVX particles until the third round of infection.

A small-scale purification of plant-produced N protein was performed. In brief, lyophilized leaf material was ground in liquid nitrogen, re-suspended and homogenized with an ultraturrax in 8 M urea, 100 mM NaH_2_PO_4_, 1 mM Tris, pH 8.0, and incubated for 1 h at RT with gentle shaking. Leaf material was finally lysed on ice by sonication at 10 Hz output (10 s each) for three times. Cell debris collected by centrifugation was discarded. The recovered supernatant was filtered (0.45 μm) and incubated for 2 h with the Ni-NTA resin. Plant N protein purification was performed by decreasing the pH (The QIAexpressionist). Protein elution was obtained at pH 4.5.

### Immunoblotting

For immunoblotting analysis of the N protein, plant extracts containing 20 μg TSP and purified protein were boiled for 3 min in SDS-loading buffer. Immunoblotting detection of the M protein was performed on plant extracts incubated at 40°C for 20’ in SDS-loading buffer. Samples were separated by 12% SDS-PAGE and transferred onto PVDF membranes (Immobilon-P, Millipore). After membrane blocking with 5% non-fat dry milk in PBS (MPBS) over night (O/N) at 4°C, membranes were incubated for 2 h at room temperature (RT) either with an anti-His_6_ monoclonal antibody (mAb; H1029-clone HIS-1, Sigma) or with specific polyclonal antibodies previously described (Carattoli et al., 2005). For N protein detection, we used a rabbit anti-N pAb, (obtained after immunization of animals with the purified His_6_-N protein produced in *E. coli*) at 1:3000 dilution. Immune complexes were revealed by 1 h incubation at RT with an anti-rabbit biotinylated secondary antibody (B8895, Sigma), at 1:5000 dilution, followed by Horseradish Peroxidase (HRP)-conjugated streptavidin (RPN1231, GE Healthcare) at 1:2000 dilution.

For M protein detection, membranes were incubated with the mouse anti-M pAb at 1:5000 dilution, followed by incubation with an anti-mouse HRP-conjugated secondary antibody at 1:10000 dilution (NA931, GE Healthcare).

For both proteins, the bound antibody was detected using the ECL Plus system (“Enhanced Chemi-Luminescence”, GE Healthcare).

### Quantitative Triple Antibody Sandwich (TAS) ELISA Assay of Leaf Extracts

The amount of N protein in the plant extracts was estimated by a quantitative triple antibody sandwich (TAS) ELISA. Symptomatic systemic leaves, deriving from 15 plants infected with pPVX-N, were collected and pooled. Three independent extractions were performed and analyzed. Microtiter plates were coated with 100 μl/well of rabbit anti-N pAb (diluted 1:3000 in PBS) for 3 h at 37°C followed by coating with 100 μl plant extract with a normalized TSP content (50 μg) for 16 h at 4°C. Wells were then blocked with 150 μl/well of 5% MPBS at 37°C for 3 h. The captured N protein was detected by incubating at 37°C for 2 h with 100 μl/well of a 1:1500 dilution in 2% MPBS of a mouse anti-N pAb (obtained after immunization of animals with the purified His_6_-N protein produced in *E. coli*, Carattoli et al., 2005) followed by incubation with 1:10000 dilution of HRP-conjugated goat anti-mouse IgG antibody. After each step, wells were washed three times with PBS + 0.1% Tween 20 and one time with PBS. Enzymatic activity was measured by adding 100 μl/well v/v H_2_O_2_/ABTS [2′, 2′-azino bis-(3-etilbenzotiazolin) sulphuric acid] (KPL Inc., Gaithersburg, MD, USA) at RT in darkness condition. The absorbance of the samples was read after 30′ at 405 nm on an ELISA microtiter plate reader. Known amounts of *E. coli*-purified N protein (0.5, 2, 5, 20, 50, 100, and 150 ng) were used as a standard. In order to give a better estimation of N protein yields in plant, the standard was diluted in *N. benthamiana* extract.

### Antigenicity Assay of the N Protein Produced in Plant with Patient Sera

A ‘multi-strip’ western blot assay was chosen to evaluate the antigenicity of the plant produced N protein. It is an immunoblotting procedure modified from Kiyatkin and Aksamitiene (2009), in which the sample, loaded in a single-well polyacrylamide gel (a standard gel where a single preparative well is cast), is blotted onto a membrane that is then cut into strips of the same width, each containing same amount of protein. Each strip is then incubated with different antibodies or sera and developed by a colorimetric assay (see Ciufolini et al., 1999; Di Bonito et al., 2002).

In our experiments, single-well 12% SDS-PAGE gels were loaded with one of the following preparations: (i) *E. coli* purified N protein (7.5 μg); (ii) plant-purified N protein (3.7 μg); (iii) plant extract from pPVX-N symptomatic leaves containing about 3.7 μg of N protein and 2 mg TSP; (iv) plant extract from pPVX201 symptomatic leaves containing about 2 mg TSP.

Samples (iii) and (iv) were prepared as following: the powder ground from pPVX-N or pPVX201 symptomatic leaves was re-suspended and homogenized by an ultraturrax in one volume (w/v) of TN buffer (25 mM Tris-HCl pH 7.2, 150 mM NaCl) containing protease inhibitors. Tissue homogenates were centrifuged at 4°C, 15000 ×*g*, for 15′. TSP was calculated by Bradford assay and precipitated by trichloroacetic acid (TCA) at 25% final concentration. After 30’ incubation in ice, samples were centrifuged, pellets were washed with cold 80% acetone, dried, re-suspended in SDS loading buffer. Gels were blotted using a semi-dry system (BIORAD) onto PVDF membranes.

After blocking with 5% MPBS, membranes were dried at RT and cut in 15 strips, 0.5 cm width. Each strip of samples (ii) and (iii) contained approximately 250 ng of the N protein (as plant-purified protein or in plant extract, respectively). As controls, strips containing 500 ng of *E. coli*-produced N protein [sample (i)], were prepared, as well as strips of plant extracts without the N protein [extract from pPVX201 symptomatic leaves, sample (iv)]. Before incubation with human sera, one strip from either plant-derived or *E. coli*-expressed N protein was incubated with the mouse anti-N pAb, as previously described (Carattoli et al., 2005), to confirm protein presence and to check that the amount of protein in the strips was sufficient for colorimetric detection.

The strips were incubated with pools of SARS sera collected during the SARS outbreak in Hong Kong in 2003 (Chan et al., 2005; 86 SARS patients pooled in seven groups) at 1:100 dilution in 3% MPBS, O/N at 4°C. Blood samples were collected with informed written consent. The study was approved by the institutional human research ethics committee (The Joint Chinese University of Hong Kong–New Territories East Cluster Clinical Research Ethics Committee).

As non-SARS controls, strips were incubated with three pools of sera from 30 patients affected by unrelated respiratory diseases (Carattoli et al., 2005). Antigen-antibodies complexes were revealed by a rabbit anti-human IgG (H + L) HRP-conjugate (Southern Biotechnology Associates, Inc, Cat.No.6145-05) diluted 1:5000 in 3% MPBS. After 1 h of incubation at RT colorimetric reaction on the strips was induced by adding 3,3′-Diaminobenzidine tetrahydrochloride substrate, DAB (Sigma D-5637) and Hydrogen peroxide.

## Results

### Expression and Purification of Plant-Derived SARS-CoV N Protein

To produce the recombinant SARS-CoV N protein, *N. benthamiana* plants (4 weeks old) were infected with the pPVX-N DNA plasmid harboring the N gene (**Figure 1A**). As a control, plants were either mock infected or infected with the wild type pPVX201 vector. While mock infected plants showed no symptoms, typical symptoms (mainly chlorotic spots) generally appeared on the inoculated leaves of plants infected with pPVX-N or pPVX201 vectors 4–5 days post inoculation (dpi). The infection spread systemically to apical leaves, where symptoms appeared 7–10 dpi.

To examine whether the N protein accumulated in infected plants, soluble protein extracts were analyzed by ELISA and immunoblotting.

**FIGURE 2:**
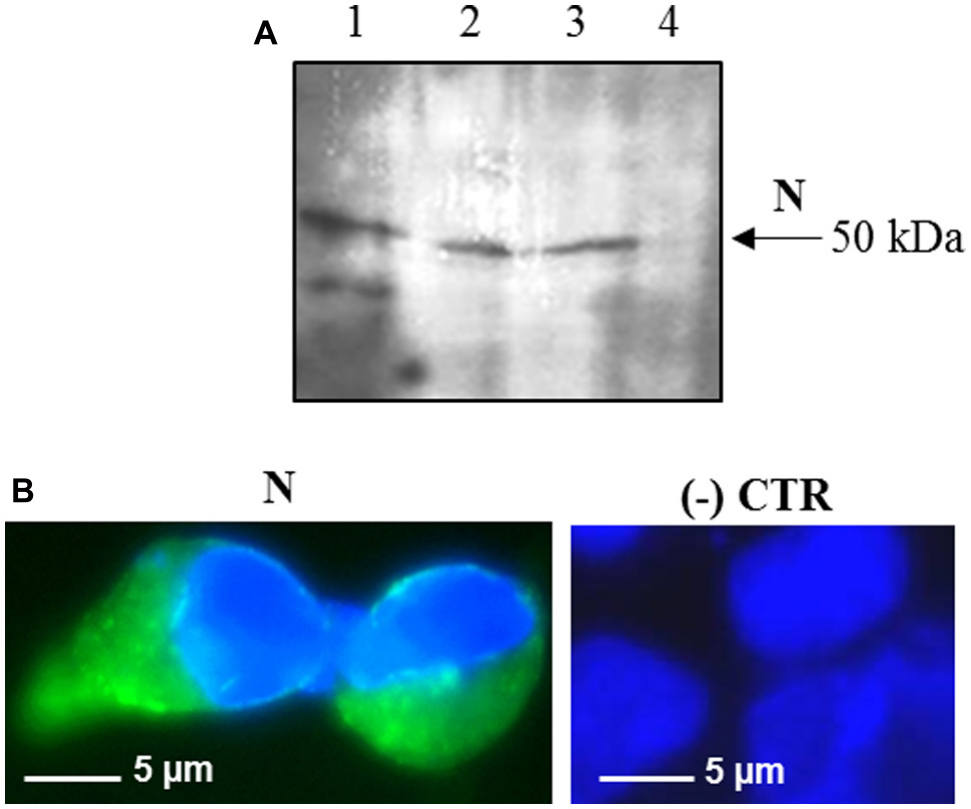
**Expression of SARS-CoV N protein in mammalian cells. (A)** Immunoblotting of total proteins extracted from HEK-293 cells transfected with the pVAX-N plasmid. For each sample, total proteins from 1 × 10^5^ cells were loaded on gel. Lane 1: N protein purified from *E. coli* under native conditions (20 ng); lanes 2, 3: HEK-293 cells, 24 h post-transfection with the pVAX-N plasmid, with or without the addition of proteasome inhibitor MG-132, respectively; lane 4: negative control, HEK-293 cells transfected with the pVAX empty vector. Immunoblotting was performed with the rabbit anti-N pAb. **(B)** Immunofluorescence of HEK-293 cells transfected with the pVAX-N plasmid (N) or with the pVAX empty vector [(-) CTR]. Immunofluorescence was performed with the rabbit anti-N pAb and nuclei were counter-stained with DAPI (100x objective, ZEISS ACHROSTIGMAT 100x/1,25 oil). Scale Bar = 5 μm.

Interestingly, after infection with the pPVX-N plasmid, protein expression corresponded to symptoms in systemic leaves in all plants analyzed (about 100). This result indicates that the construct is stable and that the recombinant virus PVX-N can spread systemically within the inoculated plant. Immunoblotting of soluble protein extracts from plants infected with pPVX-N reveals a single band of about 50 kDa in systemic leaves (**Figure 1B**), suggesting the absence of proteolysis. On the contrary, when the N protein is expressed in *E. coli*, additional bands of lower molecular mass are present (**Figure 1B**). These bands probably correspond to protein degradation, in accordance with previous work demonstrating the intrinsic instability and/or autolysis of this protein when expressed in bacteria (Mark et al., 2008). Furthermore, pPVX-N-derived extracts, stored at – 20 °C for 2 months and then thawed (**Figure 1B**), as well as extracts obtained from freeze-dried pPVX-N-infected leaves showed an intact N protein (data not shown). Immunoblotting of mammalian cells transfected with the pVAX-N plasmid also revealed the presence of a single band of approximately 50 kDa corresponding to the N protein (**Figure 2A**). Immunofluorescence revealed a cytoplasmic localization of the N protein (**Figure 2B**).

Plant extracts deriving from PVX-N-infected leaves were also used for subsequent inoculations. N protein expression was confirmed at least until the third cycle of re-infection (Supplementary Figure S1), further demonstrating the stability of the construct and of the recombinant virion.

**FIGURE 3:**
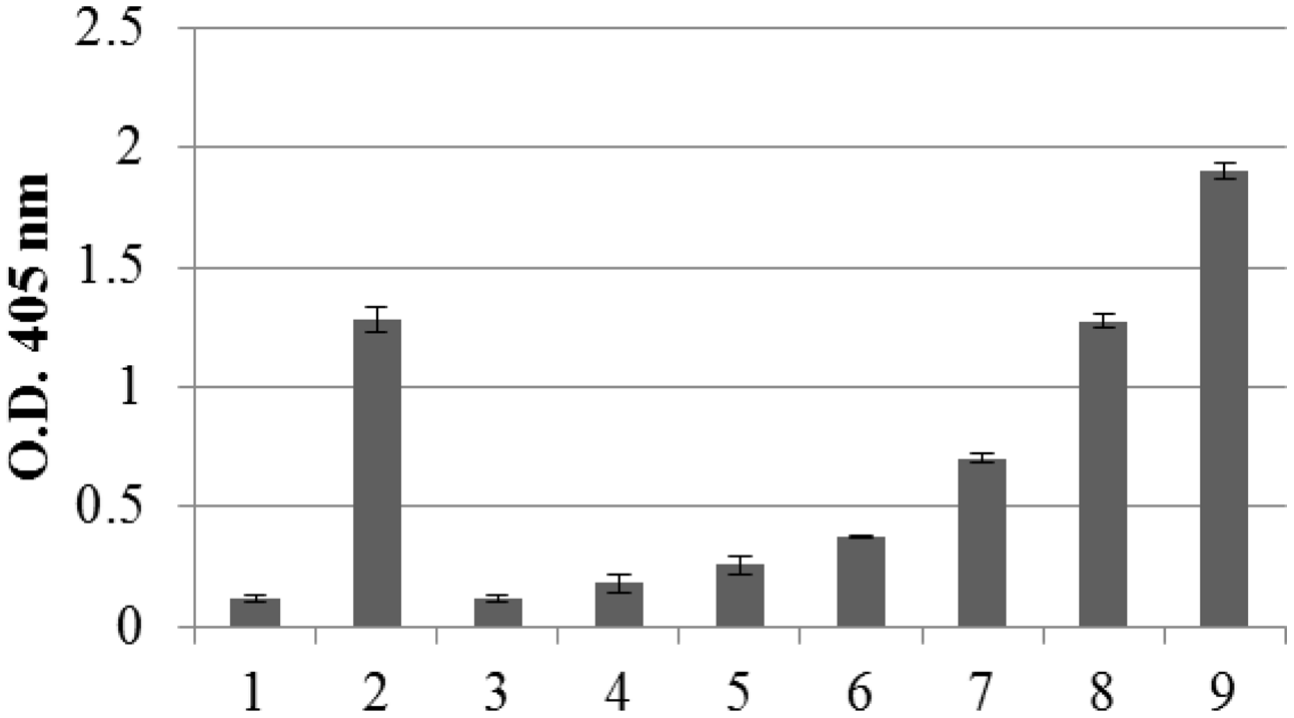
**Quantification of N protein expression in pPVX-N infected leaves by Triple Antibody Sandwich (TAS) ELISA assay**. Symptomatic pPVX-N infected systemic leaves from 15 plants were pooled and TSP (50 μg) analyzed by TAS ELISA. Data are presented as OD405 nm values (recorded at 30’) of the following samples: bar 1: pPVX201 infected systemic leaves (negative control); bar 2: pPVX-N systemic leaves; bars 3–9: purified N protein produced in *E. coli* (0.5, 2, 5, 20, 50, 100, and 150 ng, respectively) diluted in *N. benthamiana* extract. Error bars represent standard deviations of three technical replicates, i.e., three separate extractions from a pool of leaves from 15 plants (pPVX201 or pPVX-N).

The amount of recombinant N protein expressed in leaves, as measured by TAS-ELISA, was approximately 3–4 μg/g fresh leaf weight, corresponding to 0.2% TSP (**Figure 3**).

**FIGURE 4:**
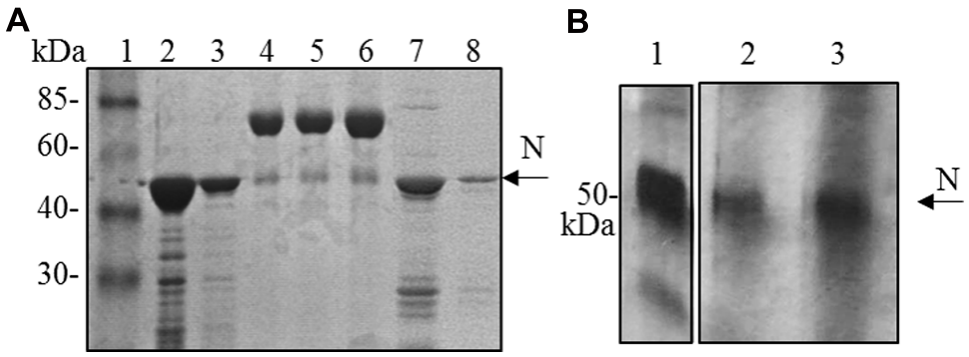
**N protein purification from *E. coli* and *N. benthamiana* plants. (A)** Coomassie blue-stained SDS-PAGE of the N protein purified from *E. coli* by affinity chromatography. Lane 1: molecular weight marker (Prestained Marker, Invitrogen); lanes 2–3: first and second elution fractions (purification performed under denaturing conditions); lane 4, 5, 6: BSA 1, 2, and 5 μg, respectively; lanes 7–8: first and second elution fractions (purification performed under native conditions). **(B)** Silver-stained SDS-PAGE of the N protein purified from *N. bethamiana* by affinity chromatography. Lane 1: N protein purified from *E. coli* under denaturing conditions (50 ng); lanes 2–3: first and second elution fractions of N protein purification from *N. benthamiana* (purification performed under denaturing conditions).

Although the N protein accumulated mainly in the soluble fraction in all the expression systems used (plant, bacteria and mammalian cells), for purification the best recovery of the N protein from *E. coli* was obtained in denaturing conditions (3 mg of protein/liter of culture under denaturing conditions versus 0.4 mg protein/liter of culture under native conditions, **Figure 4A**). Therefore, we decided to perform N protein purification in denaturing conditions also from plant tissue. We performed a small-scale purification by loading plant extracts derived from freeze-dried leaves (obtained from a pool including primary-infected and re-infected systemic leaves) on a Ni-NTA affinity purification column. In this way, we obtained yields of about 1 μg of purified N protein/g of fresh leaf weight (**Figure 4B**).

### Antigenicity of Plant-Derived SARS-CoV N Protein

The ELISA and immunoblot analysis gave a first indication of antigenic features of the plant-derived N protein. In fact, in such analysis the N protein was specifically recognized by rabbit and mouse anti-His_6_-N hyper-immune sera that had previously been shown to recognize the N protein in SARS-CoV infected cells (Carattoli et al., 2005).

**FIGURE 5:**
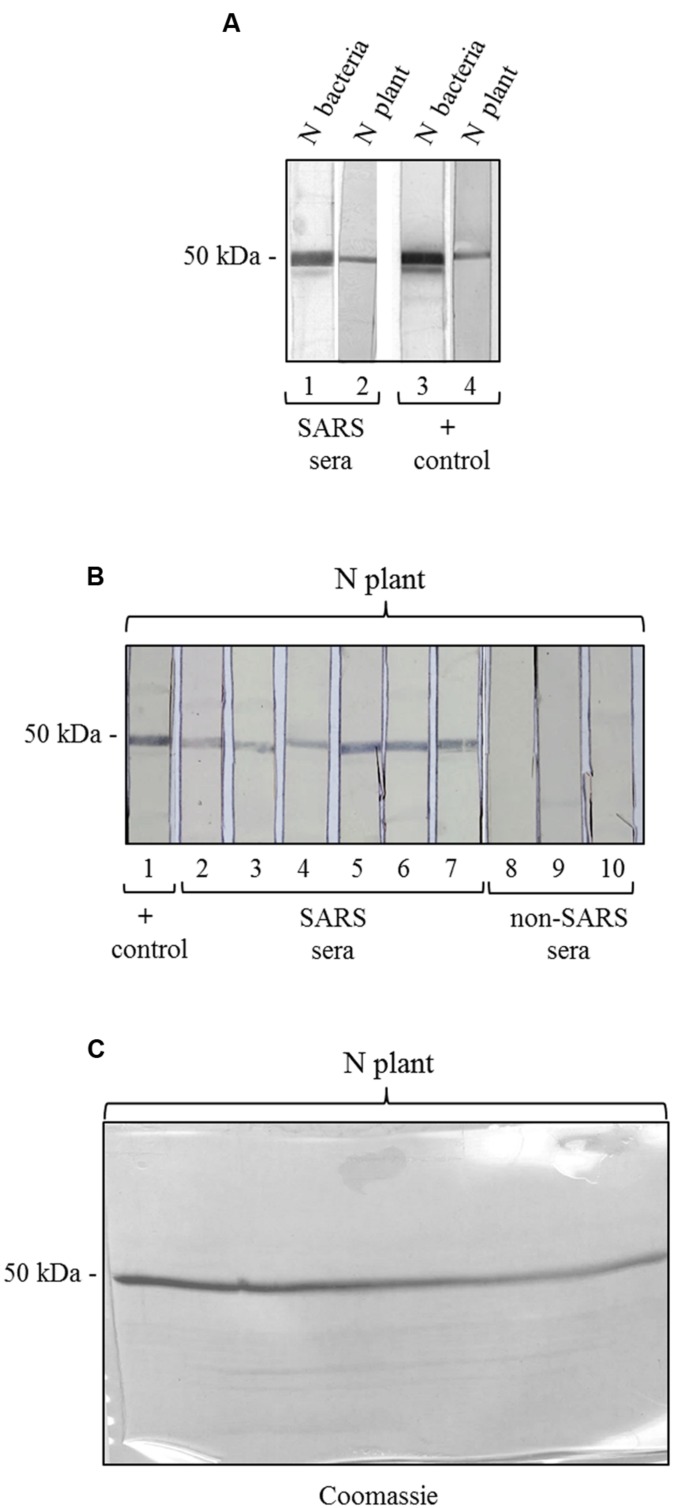
**Antigenicity of the N protein produced in plant by reaction with SARS patient sera. (A)** Reactivity of the N protein produced in plant and in *E. coli* with SARS patient sera. Strips 1, 3: purified N protein produced in *E. coli* (500 ng); strips 2, 4: purified N protein produced in plant (250 ng). Strips 1 and 2: probed with a pool of 5 SARS patient sera. Strips 3 and 4: probed with the mouse anti-N pAb (positive control). **(B)** Validation of the results shown in panel A for the N protein produced in plant. Each strip derives from the blotting of a single-well SDS-PAGE gel (showed in **C)** loaded with 3.7 μg of purified N protein produced in plant. Each strip contains approximately 250 ng of N protein. Strip 1 was probed with the mouse anti-N pAb (positive control). Strips 2–7: probed with SARS patient sera (different pools deriving from several SARS patients). Strips 6–8: probed with pools of sera deriving from patients affected by non-SARS respiratory diseases (negative control). **(C)** Replica gel of the experiment shown in panel B stained by Coomassie Brilliant Blue for loading control of the purified N protein from plant.

To further characterize the N protein expressed in plant, we analyzed its reactivity with sera from SARS patients, collected during the SARS outbreak in Hong Kong in 2003. These SARS sera were previously screened by an ELISA assay with an *E. coli*-expressed N protein (Di Bonito and Chan, data not shown). Here, to evaluate SARS-positive sera reactivity with N plant expressed protein, we used a ‘multi-strip’ western blot assay. In a first experiment, we evaluated the reactivity of the purified N protein with a pool of 5 SARS sera. As shown in **Figure 5A**, both preparations of purified N protein, from *E. coli* and from plant, are recognized by SARS sera as well as by the mouse anti-N pAb (positive control). Then, we validated the results obtained for the plant-purified N protein analyzing its reactivity with other six groups of SARS sera deriving from 86 patients and with three groups of sera from 30 patients affected by non-SARS respiratory diseases (**Figure 5B**). In this experiment, we observed that the N protein purified from plant is specifically recognized by all the groups of SARS sera analyzed, while no reactivity was observed with sera from patients affected by other respiratory diseases.

We also tested the reactivity of SARS and non-SARS sera with the unpurified N protein (soluble extract of pPVX-N symptomatic leaves) and with the extract from plant infected with the pPVX201 empty vector. A light reactivity with SARS sera was observed only for the unpurified N protein, while no reactivity was observed for the same preparation when using non-SARS sera (Supplementary Figure S2). Importantly, the pPVX201 leaf extract did not react with any human sera (Supplementary Figure S2).

To our knowledge, this is the first report of the antigenic properties of a plant-derived N protein as revealed by direct serology using SARS patient sera, suggesting its possible use for the development of SARS diagnostic assays.

### Expression of Plant-Derived SARS-CoV M Protein

We investigated the ability of plant expression systems to cope with the synthesis of M protein. We started our studies on M protein expression in *N. benthamiana* plants (4-week old) by infection with the pPVX-M plasmid harboring the *M* gene as described for the N protein. Also in this case typical symptoms generally appeared 4–5 dpi on inoculated leaves and 7–10 dpi on systemic leaves. However, M protein production was reported in just 2 plants out of 100 analyzed at detectable levels (data not shown).

**FIGURE 6:**
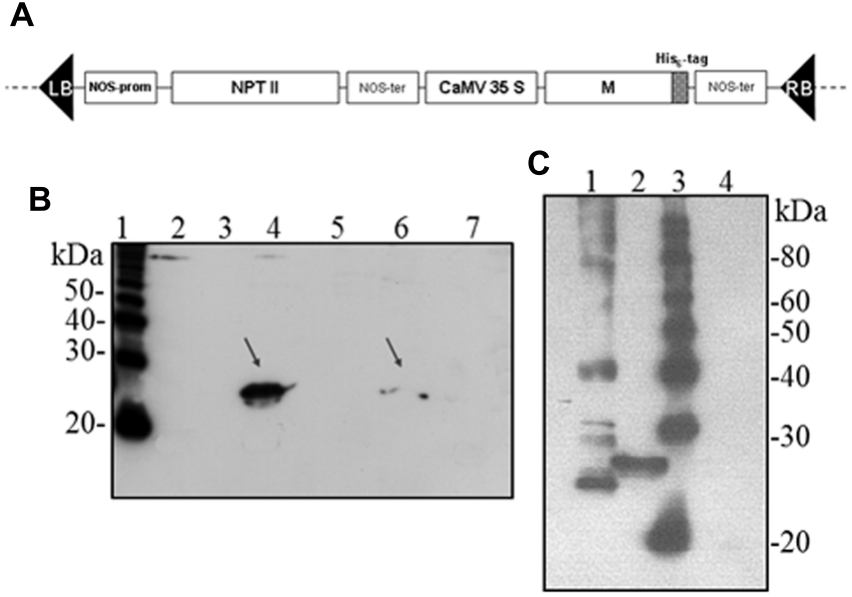
***Agrobacterium*-mediated expression of SARS-CoV M protein in *N. benthamiana* plants. (A)** Schematic representation of the construct used for agroinfiltration. CaMV 35S: cauliflower mosaic virus constitutive 35S promoter; NOS-ter: transcription terminator of the nopaline synthase gene of *A. tumefacien*; RB, LB: right and left borders; NPT II: gene for kanamycin resistance. **(B)** Comparison of M protein expression in plant by PVX-mediated infection or by agroinfiltration and solubility (western blot analysis of 40 μg TSP). Lane 1: molecular weight marker (Magic Mark, Invitrogen); lanes 2, 3: soluble and insoluble fractions, respectively, from symptomatic systemic leaves infected with pPVX-M; lanes 4, 5: soluble and insoluble fractions, respectively, from leaves infiltrated with *A. tumefaciens* C58C1/pBI-M_;_ lanes 6, 7: soluble and insoluble fractions, respectively, from leaves infiltrated with *A. tumefaciens* GV3101/pBI-M. **(C)** Comparison of M protein expression in *E. coli* or plant (western blot analysis of 20 μg TSP). Lane 1: purified M_RLV_ protein produced in *E. coli* (20 ng; purification performed under denaturing conditions); lane 2: leaves infiltrated with *A. tumefaciens* C58C1/pBI-M; lane 3: molecular weight marker (Magic Mark, Invitrogen); lane 4: negative control, leaves infiltrated with *A. tumefaciens*/pBI121 empty vector. **(B)** and **(C)**: immunoblotting performed with the mouse anti-M pAb.

Hence, we investigated the possibility to obtain the M protein by agroinfiltration. *N. benthamiana* leaves were infiltrated with *A. tumefaciens* suspensions (strains C58 and GV3101) harboring the pBI binary vector containing the *M* gene (pBI-M vector, **Figure 6A**). With this simple technology, and without the use of any post-transcriptional gene silencing suppressors, we were able to detect SARS-CoV M protein production in plant. The time course experiment revealed that the M protein is expressed mainly at 4 and 5 dpi (data not shown). For immunoblotting analysis, the M protein was extracted with an appropriate buffer (GB buffer) and, since it was previously reported that boiling causes M protein aggregation and precipitation (Lee et al., 2005), samples boiling was avoided. Interestingly, the M protein accumulated almost totally in the soluble fraction of the plant extract (**Figure 6B**). As described in the previous paragraph for the N protein, to better characterize the plant-derived M protein we worked, at the same time, on M protein expression in bacteria and mammalian cells. Due to toxic properties of M protein for *E. coli* (Carattoli et al., 2005), we performed colony selection and M protein expression under sub-optimal growth conditions (30°C). In this way, the M protein, with a mass of about 25 kDa was purified by Ni-NTA affinity chromatography in denaturing conditions, obtaining yields of about 100 μg/l culture (data not shown). DNA sequence analysis revealed the presence in the *M* gene of three spontaneous point mutations: R_13_ > K in the N-terminal domain, L_36_ > F in the first transmembrane domain and V_160_ > I in the cytoplasmic domain (M_RLV_). As the plant-derived recombinant M protein, the M_RLV_ was also specifically recognized by the mouse anti-M pAb (**Figure 6C**) that had previously validated by Immunofluorescence Antibody Assay (IFA) in SARS CoV infected Vero cells (Carattoli et al., 2005). Although we were not able to express the M protein with its original aa sequence in bacteria, the M_RLV_ protein was useful as standard for the plant-produced M protein characterization. Immunoblotting revealed that, contrary to prokaryotic cells, the plant system allowed the expression of the full-length M protein, especially by the use of the *Agrobacterium* C58C1 strain (**Figure 6B**). The M protein yield, calculated by immunoblotting using the quantified M_RLV_ protein purified from *E. coli* as standard, was estimated to be 0.1–0.15% TSP. The mobility of the plant-derived M protein in SDS-PAGE was reduced compared to the mutated bacterial form (**Figure 6C**), suggesting a higher molecular mass of plant-expressed compared to the *E. coli*- expressed M protein.

No attempts to purify the M protein from plant were done due to the expression estimates.

**FIGURE 7:**
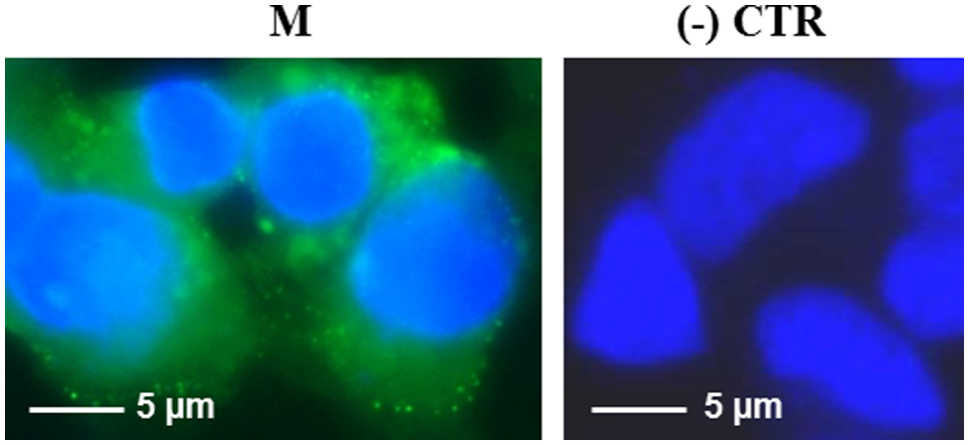
**Expression of SARS-CoV M protein in mammalian cells**. Immunofluorescence of HEK-293 cells transfected with the pVAX-M plasmid (indicated as M) or mock-transfected [pVAX empty vector, indicated as (-) CTR]. Immunofluorescence was performed with the mouse anti-M pAb and nuclei were counter-stained with DAPI (100x objective, ZEISS ACHROSTIGMAT 100x/1,25 oil). Scale Bar = 5 μm.

The M protein was also expressed in mammalian cells. Immunofluorescence on M-transfected HEK-293 cells using the mouse anti-M pAb revealed that M protein is primarily localized in the plasma membrane (**Figure 7**). Similar results were previously reported by Tseng and collaborators (Tseng et al., 2010), who described a perinuclear and plasma membrane localization of the M protein expressed in various cell lines. However, we did not observe M protein expression by immunoblotting, either in transfected cells or in the culture medium (not shown), probably because of its low expression level, as we reported in *N. benthamiana* plants by PVX-mediated infection.

## Discussion

The importance to develop effective therapeutic and preventive strategies, to be readily applied to new emergent pathogens is established by the two novel coronaviruses that have emerged in humans in the twenty-first century: severe acute respiratory syndrome coronavirus (SARS-CoV) and Middle East respiratory syndrome coronavirus (MERS-CoV). Both viruses cause acute respiratory distress syndrome and are associated with high mortality rates. There are no clinically approved vaccines or antiviral drugs available for either of these infections, thus their development represents a research priority (Graham et al., 2013).

Severe acute respiratory syndrome coronavirus was the first massive infectious disease outbreak and it still has the potential to cause large-scale epidemics in the future. The key to preventing and controlling a future outbreak of SARS is to develop rapid and specific diagnostic methods so that suspected patients can be correctly assessed. Moreover, effective and safe treatment/vaccination will be extremely important in minimizing the damage of a new pandemic. Enormous efforts have been undertaken to these purposes. The four major diagnostic methods available for SARS include viral RNA detection by RT-PCR, virus induced antibodies by immunofluorescence assay (IFA), or by enzyme linked immunosorbent assay (ELISA) of nucleocapsid protein (N) and inoculation of patient specimens in cell culture (World Health Organization [WHO], 2003 SARS: Laboratory diagnostic tests).

The SARS-CoV N protein, expressed at early stage of infection and triggering a strong antibody response by the host, is considered to be the best diagnostic target (Surjit and Lal, 2008). Plasmon resonance-based biosensors (Huang et al., 2009; Park et al., 2009) and nanowire/carbon nanotube transistors (Ishikawa et al., 2009) have been developed for the detection of SARS-CoV N protein in patient sera. Such sensors offer real-time detection of nanomolar concentrations of the protein. Nevertheless, SARS tests should also have other useful features such as cost-effectiveness and ease of operation. Moreover, combinations of antigens may be necessary to provide a definitive diagnosis of SARS in humans and susceptible animals (Roper and Rehm, 2009).

The production of recombinant N protein has been achieved in a variety of heterologous expression systems. A synthetic gene with optimized codons has been expressed in *E. coli* at high yield (Das and Suresh, 2006) but it was demonstrated that bacterially expressed N protein produces false seropositivity owing to interference of bacterially derived antigens (Leung et al., 2006; Yip et al., 2007; Surjit and Lal, 2008) or cross-reacts with antisera of human coronaviruses (HCoV-OC43 and HCoV-229E)- infected patients (Woo et al., 2004). An interesting study correlated the phosphorylation state of the N protein with its antigenicity and specificity of antibodies recognition (Shin et al., 2007). These data underline the importance of producing the recombinant protein in eukaryotic platforms such as insect cells, yeast, or plants to set up more efficient and specific diagnostic tests. To date, it was demonstrated that the N protein produced in insect cells may be useful for the development of highly sensitive and specific assays to determine SARS infection (Shin et al., 2007). Later, the N protein was transiently expressed in plant by agroinfiltration, and its antigenicity was demonstrated in mice (Zheng et al., 2009) giving a proof of concept of its use in vaccine formulations. Previously, the S1 domain of SARS-CoV S protein had been stably expressed in tomato and low-nicotine tobacco plants obtaining about 0.1% TSP (Pogrebnyak et al., 2005). The plant-derived antigen was able to induce systemic and mucosal immune responses in mice.

While previous works have primarily focused on the S and N proteins, there is growing evidence of the potential of the M antigen both as an effective vaccine and diagnostic candidate. Thus, the obtainment of the recombinant full-length M protein in an eukaryotic expression system also represents a good way to develop an effective and safe SARS-CoV vaccine. In addition to the knowledge that the M protein elicits a strong humoral responses, and that a specific humoral and cellular immune response can be obtained by co-expressing S, M and E (Lu et al., 2007), it has been demonstrated that the M protein also contains T cell epitopes (Liu et al., 2010). The availability of recombinant M protein, in combination with other viral proteins might overcome the concern about the sensitivity and the specificity of N nucleoprotein-based assay, as described when using the N and the S proteins together (Woo et al., 2004; Haynes et al., 2007; Gimenez et al., 2009). This would help the development of more efficient reagents to detect antibodies in the infected human host.

Here, we propose the use of plants as an alternative system to produce the N and M antigens that could be useful to formulate new vaccines and diagnostic assays against SARS.

Since plant transformation and regeneration of stable transformants require considerable time, we used transient expression systems (PVX and agroinfiltration) to evaluate the ability of the plant expression system to cope with the synthesis of the SARS-CoV M and N proteins.

The N and M full-length genes of the human SARS-CoV Frankfurt I isolate were cloned, without codon optimization, into different expression vectors. For the SARS-CoV N protein, we assessed the successful ectopic expression in *N. benthamiana* plants by pPVX-mediated infection (**Figure 1**). We were able to obtain the N protein in systemic leaves in most primary-infected plants as well as in 100% re-infected plants. These results demonstrate the stability of the construct, a condition not easily obtained especially when large sequences are inserted in the pPVX-derived expression cassette (the *N* gene is about 1300 bp). In fact, several studies report that the use of ‘first generation’ plant viral vectors (like the pPVX series) for the expression of proteins in plants can lead to insert elimination by natural selection over replication cycles as early as the first infection passage with a positive correlation between insert length and elimination rate (Avesani et al., 2007).

Unlike the observed prokaryotic expression pattern, no proteolysis products were detected in immunoblotting by using polyclonal sera in pPVX-N-derived extracts, fresh or stored at – 20°C for 2 months (**Figure 1B**). These data demonstrate the stability of the recombinant N protein when transiently expressed in *N. benthamiana*. The same stability was observed when the N protein was expressed in mammalian cells, even in the absence of proteasome inhibitor (**Figure 2A**).

The purified plant-produced N protein is specifically recognized by sera from Chinese SARS patients of the 2003 outbreak, and not from patients affected by unrelated respiratory diseases (**Figure 5**). This result suggests that the plant-expressed SARS-N protein is suitable in SARS diagnosis.

It is interesting to note that, when using crude plant extracts, SARS human sera reveal a weak band, corresponding to the molecular weight of the N protein, without any cross-reaction with other components of the plant extract (Supplementary Figure S2). Taken together, the results indicate that plant-derived N protein is specifically detected by antibodies of SARS patients, using an assay where the antigen-antibody complex is revealed by a colorimetric method, less sensitive but more specific and suitable for hospital clinical laboratories. It should be noted that the only information available to date about plant-derived SARS-CoV N protein antigenicity were from Zheng et al. (2009) who immunized mice and evaluated the impact on the regulation of cytokines and on the elicited IgG subclasses. Our study is the first demonstration by direct serology that plant-derived N protein is able to reveal human N-specific antibodies present in sera of SARS patients, thus providing an adequate instrument to develop a rapid, low-cost, immune-based diagnostic assay to be used as an alternative or in association to molecular diagnosis.

For the M protein, we obtained a spontaneously mutated M_RLV_ protein in *E. coli.* This allowed to overcome toxicity of the wild type protein when expressed in bacteria (Carattoli et al., 2005) and to purify the M_RLV_ protein that was then used as positive control in our experiments. As the SARS-CoV M protein is difficult to express in recombinant form, the exact structure and function of the protein are not fully elucidated (Neuman et al., 2011; Tseng et al., 2013; Siu et al., 2014).

Unlike prokaryotic cells, the plant system allowed the expression of the full-length M protein by using *A. tumefacien*s (in particular the strain C58C1), demonstrating for the first time the possibility to express the SARS-CoV M protein in plants, without the need of codon optimization or addition of any further modifications. On the contrary, by PVX-mediated infection we observed very low expression levels for the M protein, only in 2 plants out of 100 analyzed. We can speculate that membrane M protein, that already proved to be toxic in *E. coli*, may be difficult to express by using a ‘living vector’ like PVX due to interference with virus replication and/or expression/assembly of viral components, while it is tolerated by the plant when expressed by agro-infiltration.

Compared to the mutated M_RLV_ protein produced in *E. coli*, the M protein produced in plant shows a reduced electrophoretic mobility, suggesting a higher molecular mass (**Figure 6C**). The reason for this difference remains to be elucidated, but it could be due to modified residues in the M_RLV_ protein or by the presence of glycosylation in the M protein produced in the eukaryotic system (the native protein is N-glycosylated at the fourth residue).

The plant-produced M protein yields were not sufficient to perform the test with human sera. Thus, for plant-derived M protein characterization, efforts should be made in order to enhance expression yields. This includes the use of second-generation viral vectors (Gleba et al., 2007) or other implemented platforms that have been developed in the last years (Moustafa et al., 2015; Paul et al., 2015; Sack et al., 2015).

## Conclusion

Our results add further insights to the characterization of the N and M proteins and provide a proof of principle for using plants as a robust, rapid and flexible production system for protein reagents suitable to face potential recurring SARS-CoV outbreaks.

## Author Contributions

RF, PDB concept and design of the research, analysis and interpretation of data, writing and revising the article, final approval of the version to be published. OCD, SM design of the research, acquisition of data, analysis and interpretation of data, drafting and writing the article. EI acquisition of data, analysis and interpretation of data. DDM writing and revising the article critically for important intellectual content. PKSC design and revising the article critically for important intellectual content and final approval of the version to be published.

## Conflict of Interest Statement

The authors declare that the research was conducted in the absence of any commercial or financial relationships that could be construed as a potential conflict of interest.
