# Supplementary material for: Antigen Production in Plant to Tackle Infectious Diseases Flare Up: The Case of SARS
Source: Front Plant Sci. 2016 Feb 5;7:54. doi: 10.3389/fpls.2016.00054 (PMC4742786; doi:10.3389/fpls.2016.00054)
Supplement: Supplementary file 1 [file Presentation_1.PDF]

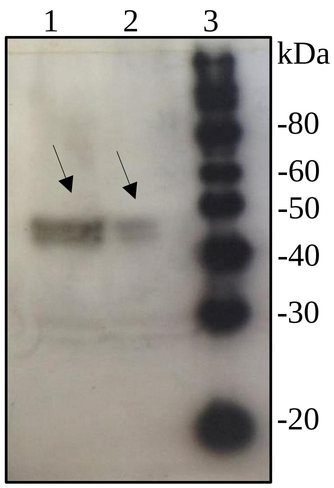

Figure S1. SARS-CoV N protein expression in *Nicotiana benthamiana* plants after secondary and tertiary pPVX-N infections

For each sample 20 µg TSP were loaded on gel.

Lane 1: pPVX-N symptomatic systemic leaves of 2th round of re-infection; lane 2: pPVX-N symptomatic systemic leaves of 3th round of re-infection; lane 3: molecular weight marker (Magic Mark, Invitrogen). Immunoblotting performed with the rabbit anti-N pAb.
