# Supplementary material for: Antigen Production in Plant to Tackle Infectious Diseases Flare Up: The Case of SARS
Source: Front Plant Sci. 2016 Feb 5;7:54. doi: 10.3389/fpls.2016.00054 (PMC4742786; doi:10.3389/fpls.2016.00054)
Supplement: Supplementary file 2 [file Presentation_2.PDF]

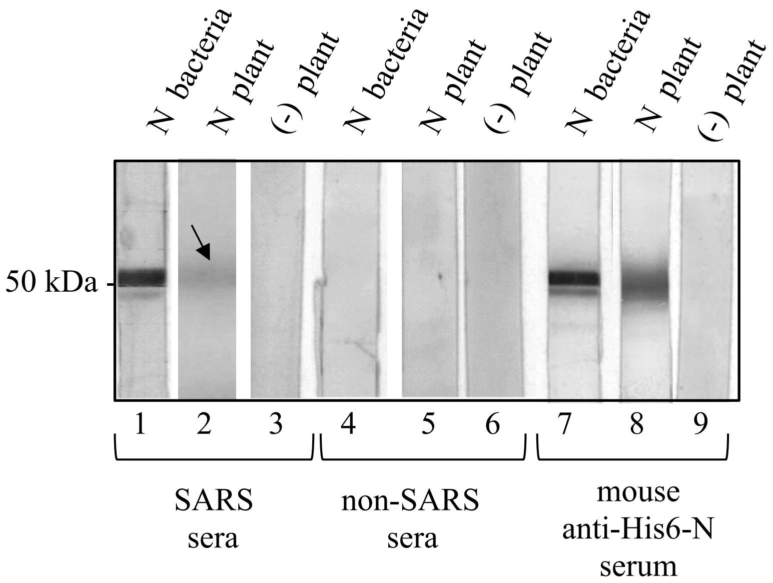

Figure S2. Reactivity of SARS patient sera with plant extract containing the N protein

Strips 1, 4, 7: purified N protein produced in *E. coli* (500 ng); strips 2, 5, 8: crude extract from pPVX-N systemic leaves (pool of 15 plants) containing 250 ng of plant-derived N protein; strips 3, 6, 9: crude extract from empty vector pPVX201 systemic leaves (pool of 15 plants).

Strips 1, 2, 3 were probed with SARS patient sera

Strips 4, 5, 6 were probed with non-SARS patient sera (negative control)

Strips 7, 8, 9 were probed with a mouse anti-His6-N serum (positive control).

Results shown in this figure are representative of 5 independent experiments (performed with pools of 5 SARS- or 3 non-SARS-patients sera).
